# The efficacy and safety of once-daily quetiapine extended release in patients with schizophrenia switched from other antipsychotics: an open-label study in Chinese population

**DOI:** 10.1186/s12888-014-0378-5

**Published:** 2015-01-22

**Authors:** Pei-Yin Pan, Meei-Shyuan Lee, Chin-Bin Yeh

**Affiliations:** Department of Psychiatry, Tri-Service General Hospital, National Defense Medical Center, No.325, Sec.2,Chenggong Rd., Neihu Dist., Taipei City, 114 Taiwan; School of Public Health, National Defense Medical Center, No.161, Sec. 6, Minquan E. Rd., Neihu Dist., Taipei City, 114 Taiwan

**Keywords:** Schizophrenia, Quetiapine XR, Antipsychotic agents, Drug switching, Chinese population

## Abstract

**Background:**

Non-adherence to antipsychotic medication in schizophrenic patients is common and associated with symptom relapse and poorer long-term outcomes. The risk factors for treatment non-adherence include dosing frequency and complexity. Besides, slower dose titration in an acute schizophrenic episode may lead to attenuated efficacy. Therefore, the convenient dosage regimen and rapid initiation scheme of quetiapine extended release (XR) were expected to provide better effectiveness and promote adherence in patients with schizophrenia. This study was implemented to assess the efficacy and safety of once-daily quetiapine XR in schizophrenic patients with switched from other antipsychotics which were suboptimal due to insufficient efficacy or tolerability.

**Methods:**

This was a 12-week, open-label study conducted in the Chinese population in Taiwan.

Patients who had a score of 4 (moderate) or greater on any of the 7 items of the Positive and Negative Syndrome Scale (PANSS) Positive Symptom Subscale and needed to switch from previous antipsychotics were recruited. Quetiapine XR was administered at 300 mg on day 1, 600 mg on day 2 and up to 800 mg after day 2. From day 8 until the end of the study, the dose of quetiapine XR was adjusted within 400-800 mg per day, depending on the clinical response and tolerance of the patients. The variable of the primary outcome was the change from baseline to Week 12 in PANSS total and subscale scores. Secondary outcome was the baseline-to-endpoint difference in the Clinical Global Impression-Severity (CGI-S) scores of the participants.

**Results:**

Sixty-one patients were recruited and 55.7% of them completed the study. The mean changes in the PANSS total score and CGI-S score showed significant improvement (−18.4, p < .001 and −1.0, p < .001, respectively). Four patients (6.7%) experienced adverse events including headache, exacerbation of psychosis and dysuria. The use of concomitant anticholinergics decreased from 15.0% to 8.3%.

**Conclusions:**

The results of our investigation implicated that quetiapine XR was an effective and well tolerated alternative for Chinese schizophrenic patients with previous suboptimal treatment. Future large-scale studies are warranted to validate our results.

**Trial registration:**

ClinicalTrials.gov ID NCT02142556. Registered 15 May 2014.

## Background

Antipsychotics have long been established as a necessary part of pharmacotherapeutic interventions in both acute and long-term treatment of schizophrenia to control the disturbed symptoms, reduce the risk of harm, and help the patients to regain their premorbid level of functioning [1–4]. Non-adherence to antipsychotic medication in schizophrenic patients has also been considered to be a crucial contributor to symptom relapse and poorer long-term outcomes [5–7]. However, it is common for patients with schizophrenia to have low adherence to antipsychotics [8–10] and it had been reported that almost half of schizophrenic patients took less than 70% of the expected prescribed doses [11]. The risk factors for treatment non-adherence include poor therapeutic response, adverse events related to antipsychotic medication, impaired insight, comorbid substance abuse, negative attitude towards treatment, and dosing frequency and complexity [11–14]. Although the current atypical antipsychotics show comparable efficacy and better tolerability [1,2], it is still challenging in clinical practice to improve the treatment-adherence in schizophrenic patients.

For acute schizophrenic episode, it is important to provide treatment in order to achieve an early response within the first several days [15,16]. Early improvement is associated with better subsequent symptom response and less illness chronicity [17,18]. To reach the goal of prompt symptom reduction, rapid dose escalation of antipsychotics had been recommended as a beneficial treatment strategy for acutely ill schizophrenic patients [16,19–23]. On the other hand, slower dose titration in an acute schizophrenic episode may lead to attenuated efficacy [24,25]. Accordingly, the administration of the well tolerated antipsychotics which could be rapidly titrated to target dosage is critical for acute schizophrenic patients.

Extended-release quetiapine fumarate (quetiapine XR) is a formulation with similar bioavailability to immediate-release quetiapine fumarate (quetiapine IR) [26]. Quetiapine XR also achieved comparable mean plasma concentrations as quetiapine IR, which was recommended two divided doses regimen, during a 24 h dosing interval when administered once daily [26]. Besides, the milder sedative profile of quetiapine XR [27] may be more suitable for fast initiation than quetiapine IR, which had been reported that more patients experienced somnolence and postural hypotension with the treatment of rapid dose escalation [28]. Therefore, this convenient dosage regimen and rapid initiation scheme with quetiapine XR were expected to provide better effectiveness and promote adherence in patients with schizophrenia [29,30]. It has been demonstrated that quetiapine XR is efficacious and safe in treating acute schizophrenic episodes [31,32] and in preventing symptom relapse [33,34]. In addition, two previous trials reported positive outcomes of switching to quetiapine XR from other antipsychotics in patients with suboptimal prior pharmacological treatment [35,36]. However, there was still a paucity of clinical investigation about the efficacy and tolerability of quetiapine XR in a Chinese population with schizophrenia. For the significant lower prevalence rate of schizophrenic patients [37,38] and different metabolism of psychotropic agents [39,40], the Chinese should be considered as a specific ethnic population in the investigation of the effectiveness of antipsychotics. Here we implemented a 12-week, open label study to assess the clinical benefit of switching to quetiapine XR in the Chinese schizophrenic patients in Taiwan who were dissatisfied with their ongoing antipsychotic therapy.

## Methods

### Patients

The participants who were aged from 20 to 65 years and met the diagnostic criteria for schizophrenia according to the Diagnostic and Statistical Manual of Mental Disorders, fourth edition, text revision (DSM-IV-TR) were eligible for recruitment in this clinical trial. All of them were screened at the psychiatric outpatient or acute ward of the principal investigator’s affiliation which was a medical university hospital. The subjects had a score of 4 (moderate) or greater on any of the 7 items of the Positive and Negative Syndrome Scale (PANSS) Positive Symptom Subscale and needed to switch from previous antipsychotics due to insufficient efficacy or tolerability (intolerable adverse events reported by the patient or observed by the principal investigator). Any of the following was regarded as a criterion for exclusion from the study: (1) any DSM-IV-TR Axis I disorder other than schizophrenia, except comorbid obsessive-compulsive disorder, anxiety disorder, eating disorder or impulse control disorder if they had been stable and these had not been the primary focus of treatment over the previous 6 months; (2) an imminent risk of suicide or a danger to self or others; (3) pregnancy or lactation; (4) intolerance or lack of response to quetiapine IR; (5) use of cytochrome P450 3A4 inhibitors or inducers in the 14 days preceding enrollment; (6) administration of a depot antipsychotic injection within one dosing interval before recruitment; (7) unstable or inadequately treated medical illness as judged by the investigator. Before recruitment, all of the patients provided written informed consent after the study had been thoroughly explained to them. The study was approved by the Institutional Review Board of Tri-Service General Hospital, National Defense Medical Center, Taipei, Taiwan.

### Study design

**Figure 1:**
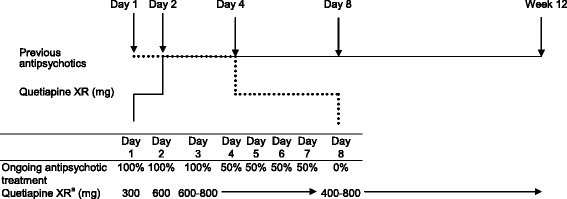
**The study design and schematic diagram of switching from other antipsychotics to quetiapine XR in this investigation.** ^a^After day 2, the dosage of quetiapine XR was allowed up to 800 mg. From day 8 until week 12, the dose of quetiapine XR was adjusted within the effective dose range of 400 mg to 800 mg per day.

This study was a 12-week, open-label investigation conducted in the Chinese population in Taiwan (November 2008 to March 2012). The treatment was initiated with a 7-day cross-titration period. Previous antipsychotic medication was maintained at the original dose from day 1 to day 3; then reduced to 50% of the original dose from day 4 to day 7 and discontinued on day 8. Meanwhile, the patients started the study medication with a daily dose of 300 mg on day 1, 600 mg on day 2 and up to 800 mg after day 2. From day 8 until the end of the study (treatment period), the dose of study medication was adjusted within the effective dose range of 400 mg to 800 mg per day, depending on the clinical response and tolerance of the patient. The administration of any other antipsychotics or psychoactive medications was prohibited during the treatment period. Patients treated with quetiapine IR preceding enrollment were direct switched to the equivalent dose of quetiapine XR. The dose and treatment regimen of this study are shown in Figure 1.

### Efficacy assessments

The variable of the primary endpoint was the change from baseline to Week 12 in PANSS total and subscale scores. Patients were evaluated at other three visits with PANSS during the study period, including week 1, week 4 and week 8. Another efficacy endpoint was the difference from baseline to the end of the study in the Clinical Global Impression-Severity (CGI-S) scores of the participants.

### Safety assessments

The occurrence and severity of adverse events (AEs) was recorded throughout the study to assess the tolerability of quetiapine XR, including AEs spontaneously reported by the patients or observed by the staff after interviewing the patient. We used Abnormal Involuntary Movement Scale (AIMS), Barnes-Akathisia Rating scale (BARS) and Simpson-Angus Scale (SAS) to evaluate the extrapyramidal symptoms (EPS) associated with the previous antipsychotics or quetiapine XR. The use of anticholinergic medications in each patient during the treatment period was also recorded.

Each patient’s vital signs and body weight were measured at screening and at every scheduled visit (week 1, 2, 4, 8, 12). The electrocardiogram (ECG) and laboratory measurements including hematology and glycosylated hemoglobin (HbA1c) were performed at enrollment and at week 12.

Adherence to the study medication was evaluated by measuring the difference between the dosage dispensed and the tablets returned at each scheduled visit. Patients who took more than 85% dosage of the study drugs as prescribed were considered to be treatment adherent.

### Statistical analysis

The intention-to-treat (ITT) population, consisting of all patients who took at least one dose of the study medication and had at least one evaluation for the primary efficacy endpoint after the cross titration period, was used for the efficacy and safety assessment. Subgroup analyses based on the reasons for switching and the previous antipsychotic treatment were also performed. A last observation carried forward (LOCF) approach was applied to handle the missing values for the efficacy analysis. To compare the baseline characteristics between the two subgroups with different reasons for enrolling, the chi-square test or Fisher’s exact test were used for categorical variables and Student’s t test for continuous variables. For the primary and the other efficacy endpoints, a two-way ANOVA with one-way repeated was used to analyze the mean change in PANSS and CGI-S scores between groups. A p value of less than 0.05 was considered to be statistically significant. All statistical analyses were performed with SPSS software version 17 (SPSS Inc., Chicago, IL, USA).

## Results

### Patient disposition and characteristics

**Figure 2:**
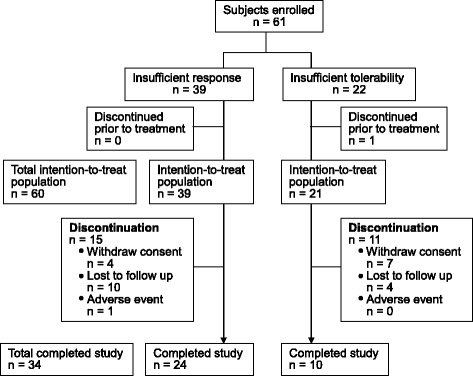
**Patient disposition during the study.**

A total of 61 patients (45 patients from the psychiatric outpatient and 16 patients from the acute ward) were recruited in the study and one discontinued before receiving the study medication. The 60 patients who took at least one dose of quetiapine XR were included in the intention-to-treat (ITT) population. Among them, 39 patients (65.0%) were recruited because of insufficient response and 21 patients (35.0%) because of insufficient tolerance to their original antipsychotics. During the 12-week follow-up period, 26 patients discontinued the study prematurely. The reasons were lost to follow up (14 patients), consent withdrawal (11 patients) and adverse events (1 patient). Thirty-four patients (55.7%) completed the study (Figure 2).

In terms of the patients’ previous antipsychotics, 15 patients (25.0%) received risperidone (2.99 ± 1.44 mg [mean daily dose ± standard deviation]), 11 (18.3%) quetiapine IR (445.45 ± 103.57 mg), 9 (15.0%) sulpiride (511.11 ± 105.41 mg), 6 (10.0%) amisulpride (480.00 ± 228.04 mg), 5 (8.3%) olanzapine (10.63 ± 7.18 mg), 5 (8.3%) aripiprazole (8.50 ± 3.35 mg), 4 (6.7%) ziprasidone (90.00 ± 50.33 mg) and 3 (5.0%) clozapine (62.50 ± 45.07 mg). One patient received aripiprazole and clozapine combination therapy; one received no antipsychotics before entering the trial and two did not report their prior antipsychotics.

The patients’ baseline demographics and clinical characteristics of the two subgroups were generally equivalent except that the patients who switched because of insufficient tolerance were younger than those recruited because of insufficient response (33.3 years vs. 40.5 years, *t* = 2.595, p = .012) (Table 1).

**Table 1:** **Patient demographics and clinical characteristics at baseline**

	**Overall (** ***n*** **= 60)**	**Insufficient response (** ***n*** **= 39)**	**Insufficient tolerability (** ***n*** **= 21)**
Gender, *n* (%)			
Male	37 (61.7)	22 (56.4)	15 (71.4)
Female	23 (38.3)	17 (43.6)	6 (28.6)
Age, years (SD)^c^	38.0 (10.7)	40.5 (10.5)*	33.3 (9.9)*
Marital status, *n* (%)^a^			
Single	42 (70.0)	25 (64.1)	17 (81.0)
Married	11 (18.3)	9 (23.1)	2 (9.5)
Divorced	4 (6.7)	3 (7.7)	1 (4.8)
Widowed	2 (3.3)	2 (5.1)	0 (0.0)
Employment status, *n* (%)^a^			
Employed	12 (20.0)	8 (20.5)	4 (19.0)
Student/Retired	4 (6.7)	1 (2.6)	3 (14.3)
Unemployed	43 (71.7)	30 (76.9)	13 (61.9)
Psychiatric history			
Disease duration, years (SD)	12.5 (9.3)	13.2 (9.6)	11.3 (8.8)
Number of schizophrenic episodes over lifetime, *n* (SD)	3.6 (2.7)	3.2 (2.1)	4.3 (3.5)
Presence of family members with schizophrenia, *n* (%)	9 (15.0)	4 (10.3)	5 (23.8)
Psychoactive medication at base, *n* (%)			
Any antipsychotics	59^b^ (98.3)	38^b^ (97.4)	21 (100.0)
Risperidone	15 (25.0)	8 (20.5)	7 (33.3)
Quetiapine IR	11 (18.3)	8 (20.5)	3 (14.3)
Sulpiride	9 (15.0)	8 (20.5)	1 (4.8)
Amisulpride	6 (10.0)	3 (7.7)	3 (14.3)
Aripiprazole	5 (8.3)	3 (7.7)	2 (9.5)
Olanzapine	5 (8.3)	3 (7.7)	2 (9.5)
Ziprasidone	4 (6.7)	3 (7.7)	1 (4.8)
Clozapine	3 (5.0)	1 (2.6)	2 (9.5)
Mean (SD) baseline scores			
PANSS	84.4 (17.7)	85.9 (17.5)	81.7 (18.2)
CGI-S	4.7 (0.7)	4.7 (0.7)	4.7 (0.7)

^a^One patient with insufficient tolerance had no reported marital status and employment status.

^b^One patient with insufficient response had received no antipsychotics before entering the trial; Two patients did not report their previous antipsychotics.

*p < .05, ^c^t test = 2.595, p = .012

### Treatment

The mean number of days of exposure to quetiapine XR was 56.63 ± 35.18 (mean ± standard deviation) for all patients and there was no significant difference between the two subgroups. The mean daily dose of quetiapine XR was 562.50 ± 177.81 mg, which was comparable between the two populations. During the follow-up period, a total of 52 patients (86.7%) were considered to be treatment adherent and 4 patients took less than 70.0% dosage of the study drugs as ordered. The mean adherence rates between the two subgroups were similar.

### Efficacy

**Figure 3:**
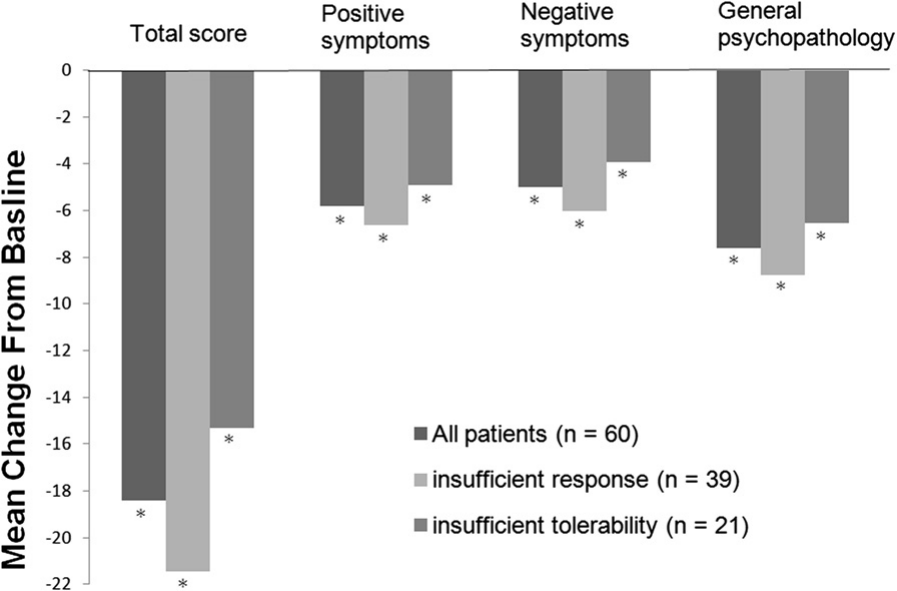
**Changes in PANSS total and subscale scores from baseline to week 12 in patients with schizophrenia receiving the treatment of switching to quetiapine XR.** The change in PANSS total and subscales score in all patients shown is based on estimated marginal means. *p < .05.

**Figure 4:**
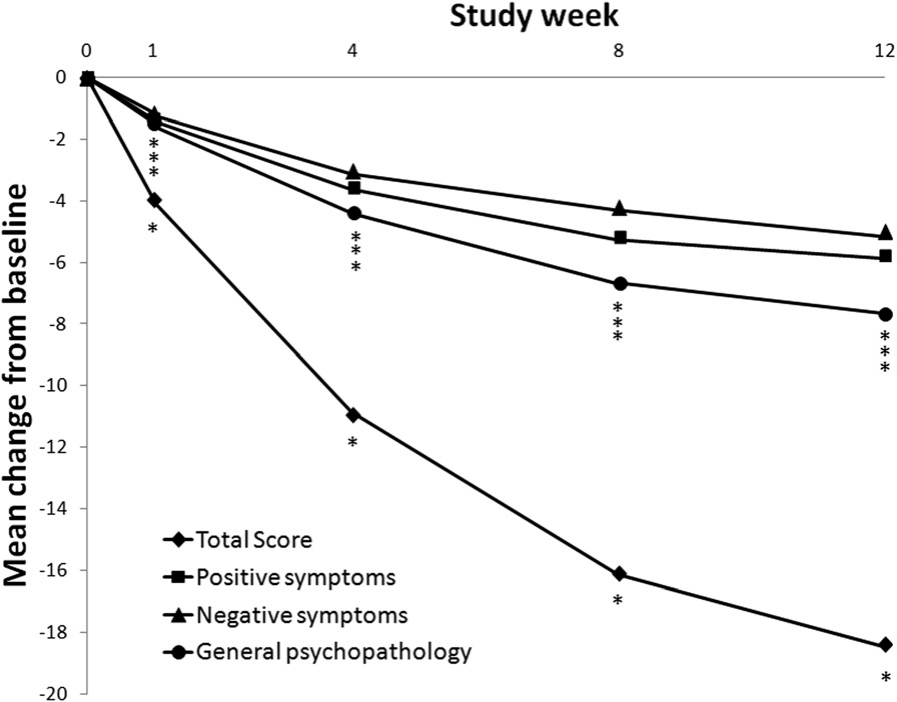
**Changes in PANSS total score from baseline to W1, W4, W8 and W12 in patients with schizophrenia after switching to quetiapine XR.** The change in PANSS total score in all patients shown is based on estimated marginal means. *p < .05.

The overall population’s PANSS total score and subscale scores were significantly improved at the week 12 evaluation. The mean changes from baseline to the end of the study (based on estimated marginal means) in PANSS total and the positive, negative and general psychopathology subscale scores were −18.4 (95% CI −22.6, −14.2; p < .001), −5.8 (95% CI −7.1, −4.4; p < .001), −5.0 (95% CI −6.4, −3.6; p < .001) and −7.6 (95% CI −9.6, −5.7; p < .001), respectively (Figure 3). Figure 4 showed the time response curve according to the improvement of PANSS total score from baseline to each visit.

The patients subdivided according to the reasons for switching also showed significant reductions in PANSS total and subscale scores with no statistical difference between the two subgroups. When we analyzed the efficacy of quetiapine XR based on the patients’ previous antipsychotic treatments, there was also a significant improvement in PANSS total and subscale scores irrespective of their prior medication. The mean changes in PANSS total scores in patients treated with different antipsychotics before switching were risperidone −17.7 ± 13.9 (mean ± standard deviation), quetiapine IR −27.6 ± 16.5, sulpiride −24.9 ± 16.4 and other antipsychotics −24.6 ± 14.4.

There was a significant improvement in CGI-S score in the overall population (mean change −1.0; 95% CI −1.3, −0.8; p < .001), the patients switching for insufficient response (mean change −1.2; 95% CI −1.6, −0.9; p < .001), and the patients recruited due to insufficient tolerability (mean change −0.9; 95% CI −1.3, −0.4; p < .001).

### Tolerability

#### AEs

During our study, quetiapine XR was well tolerated without severe AEs. Four patients (n = 4/60, 6.7%) experienced AEs of mild to moderate severity. One patient had headaches, two patients showed exacerbation of psychosis, and the other patient had difficulty in urination that she was discontinued from study. There were no deaths during the study.

#### EPS

Throughout the treatment period, quetiapine XR was found well tolerated by all patients receiving the medication. Only one patient was noted to have akathisia rated 1 on all four items of the BARS and muscle rigidity rated 1 on the item head dropping of SAS at week 4. Her symptoms resolved spontaneously without administration of any medication. Except for the patient described above, there were no obvious objective or subjective extrapyramidal symptoms observed during the treatment with quetiapine XR. The proportion of patients taking concomitant anticholinergics was 15.0% on day 1 and decreased to 8.3% at the end of the study.

#### Laboratory data, vital signs and physical findings

All patients’ pulse rates, blood pressure, hematology, HbA1c and ECG parameters showed no significant changes from enrollment to week 12 assessments. The patients’ baseline mean body mass index (BMI) was 25.4 ± 3.7 kg/m^2^ with 30.0% of the patients overweight (BMI ≥ 24) and 30% obesity (BMI ≥ 27). (according to the adjusted definition of the Health Promotion Administration, Ministry of Health and Welfare, Taiwan.) The mean change in all patients’ body weight from baseline to the end of the study was 0.0 kg. The patients’ mean BMI at week 12 was 25.4 ± 3.7 kg/m^2^ with 31.7% of the patients overweight and 28.3% obesity. No patient reported more than a 7.0% change in body weight.

## Discussion

Consistent with the previous study that the response in the first three days predicted the symptoms remission 4 weeks later in the patients with schizophrenia [41], our study demonstrated the effectiveness and good tolerability of the extended release (XR) fumarate of quetiapine administered with forced dosage titration from 300 to 800 mg within the first three days in a Chinese population after treatment with a 12-week study period. Our results showed that the patients’ PANSS total and each subscale score showed significant improvement from baseline irrespective of the reasons for enrollment in the study or prior antipsychotics. A significant reduction was also observed in the mean change from baseline in CGI-S scores. These findings provided supporting evidence that quetiapine XR was an effective therapeutic alternative for acute schizophrenic episodes. Our data was consistent with that of two previous multicenter clinical trials conducted in countries other than Taiwan [35,36], and suggested that quetiapine XR demonstrated comparable effectiveness in Chinese schizophrenic patients.

The titration scheme for quetiapine XR in this study reached a therapeutic dose within three days through initiation of therapy at a higher dose. The switching paradigm had been adopted in other studies [32,35,36] and also showed benefit in the present investigation. It has been found that the extended release formulation resulted in similar mean plasma levels but decreased fluctuations when compared to immediate release quetiapine [26,29]. That may improve tolerability and allow rapid titration to the target regimen in order to achieve an early response which was considered to be a predictor for the subsequent symptom improvement and a better outcome [17,42]. Besides, the advantages of this switching paradigm also included a reduction of the risk of additive side effects by shortening the period of cross-tapering [43]. Furthermore, the simplified dosing regimen of taking medication only once daily was thought to correlate with potentially better adherence [13,44–46] and a beneficial impact on long-term functional outcomes [5].

Our results indicated that quetiapine XR was generally well tolerated by participants, even in the first week when they underwent rapid dosage escalation to a therapeutic dose. It may be attributed to quetiapine XR’s pharmacokinetics which skip the high peak plasma concentration found in quetiapine IR [26] and therefore reduce the risk of side effect. This suggested that quetiapine XR might be more beneficial to patients with an acute schizophrenic episode since the high loading dose might have faster efficacy but have a lower incidence of adverse events such as postural hypotension, somnolence and dizziness [31] which quetiapine IR was often recommended to be slowly titrated up to avoid the occurrence [27,28,47]. Meanwhile, regarding the treatment related EPS, only one patient had transient akathisia and muscle rigidity which resolved spontaneously later. It was also noted that the percentage of concomitant anticholinergics use in all subjects reduced at the end of the study. These findings suggested that quetiapine XR improved the patients’ motor symptoms after switching from other antipsychotics. In our study, the metabolic disturbance associated with quetiapine XR was thought to be minimal since there was no significant weight gain and increased HbA1c from baseline to the week 12 evaluation. Compared to the previous literature of pooled safety data from other multicenter studies of quetiapine XR in acute schizophrenia [31], our results suggested that the tolerability of quetiapine XR was well in Chinese schizophrenic patients although the findings required further studies with a larger sample size to validate.

There are several limitations to the current study. Initially, it was a single-arm investigation without a comparative group for the evaluation of a possible placebo response and may confine the interpretation of the results [48]. Besides, the open-label algorithm in which the investigator was aware of the treatment may have influenced the assessment of outcome and led to detection bias [49,50]. Meanwhile, some participants switching for insufficient response didn’t take full dosage of their original antipsychotics before recruitment and this should be considered as one of the possible reason for the suboptimal efficacy of the prior treatment. Other limitations include the small sample size of the study leading to insufficient power, and the relatively short follow-up period compared to a previous trial [36] so that the results can only be generalised within 12 weeks. An additional limitation was that we did not measure changes in the patients’ metabolic parameters such as fasting plasma glucose, lipid profile and waist-circumference in our investigation. Therefore, we had difficulty in assessing the impact of the common metabolic side effects associated with atypical antipsychotics such as dyslipidemia [51] on the participants after the treatment of switching to quetiapine XR.

## Conclusions

The results of this study indicated that quetiapine XR was an effective and well tolerated alternative for Chinese schizophrenic patients with previous suboptimal treatment. Quetiapine XR administered once daily reached a therapeutic level earlier through initiation of a higher dose and rapid titration provided an advantage in making the successful switch from other antipsychotics. Further studies with a larger sample size and a longer follow-up period in the treatment of patients with schizophrenia will be required to verify the findings of this study.
